# A systematic review of real-world evidence on the clinical relevance, characterization, and utility of *CYP2D6* biomarker testing

**DOI:** 10.3389/jpps.2025.14708

**Published:** 2025-08-29

**Authors:** Patrick Rodriguez, Emma Kikerkov, Nora Emmott, Christine Y. Lu, Rachele M. Hendricks-Sturrup

**Affiliations:** ^1^ Duke-Robert J. Margolis, MD, Institute for Health Policy, Washington, DC, United States; ^2^ Department of Population Medicine, Harvard Pilgrim Health Care Institute and Harvard Medical School, Boston, MA, United States; ^3^ Kolling Institute, Faculty of Medicine and Health, The University of Sydney and the Northern Sydney Local Health District, Sydney, NSW, Australia; ^4^ School of Pharmacy, Faculty of Medicine and Health, The University of Sydney, Sydney, NSW, Australia

**Keywords:** electronic health records, pharmacogenomics, precision medicine, *CYP2D6*, biomarker testing, claims data, real‐world data, real‐world evidence

## Abstract

Pharmacogenomic (PGx) research investigates how an individual’s genetic make-up impacts their drug metabolism. PGx testing can therefore inform therapeutic decision-making, especially as compelling evidence develops over time to substantiate its clinical and personal utility across a range of therapeutic areas. PGx biomarker *CYP2D6*, in particular, is widely implicated in drug metabolism and across several therapeutic areas. Real-world evidence (RWE) derived intentionally using electronic health record (EHR) and insurance claims data presents an opportunity to explore clinical-behavioral outcomes and implementation barriers and facilitators for PGx testing in real-world clinical settings. In this systematic review, we explored these areas with a focus on PGx biomarker *CYP2D6*, investigating drug-gene pairs with strong evidence (Level A, Final classification by the Clinical Pharmacogenetics Implementation Consortium [CPIC]). Across 25 studies that met our study inclusion criteria, nine (9) drug-gene pairs that met the CPIC Level A, Final, strong evidence category for *CYP2D6* were described. Overarching qualitative themes across studies were 1) variation in CYP2D6 biomarker testing and interpretation, and 2) PGx test implementation and data considerations. CYP2D6-drug pairs were reported across four therapeutic areas (analgesia [n = 21], psychiatry [n = 17], oncology [n = 7], gastroenterology [n = 6]) with the two most researched drugs being codeine (n = 21) and tramadol (n = 18). Six (6) of 25 articles reported PGx clinical outcomes, considered to be a “measurable change in symptoms, overall health, ability to function, quality of life, or survival outcomes” in relation to PGx testing. Special EHR and claims data considerations for future work include but are not limited to addressing inconsistent phenotype categorizations (i.e., natural genotype versus phenoconversion); lack of reliable racial, ethnic, and genetic ancestry data within EHR and claims data sources; and data inoperability issues between PGx test results and EHRs.

## Introduction

*CYP2D6* is a key enzyme and biomarker involved in the metabolism of may commonly prescribed medications, particularly in older adults [1–3]. A large and growing body of evidence demonstrates the clinical importance of *CYP2D6* in precision medicine research and practice. The Clinical Pharmacogenomics Implementation Consortium (CPIC), a pharmacogenomics (PGx) organization with implementation guidance on *CYP2D6*, reviews evidence around the implications of different genes to adversely affect drug metabolism. To date, CPIC has assigned four evidence levels to drug-gene pairs: A, B, C, and D [4]. Drug-gene pairs assigned to “CPIC Level A, Final” are accompanied or followed by in-depth reviews of evidence to guide the practice of prescribing/de-prescribing based on PGx biomarker status. CPIC recommends prescribing action for Level A medications and that alternative medications are likely safe and effective. Currently, ten *CYP2D6*-drug pairs are assigned to CPIC Level A, Final [5].

**FIGURE 1:**
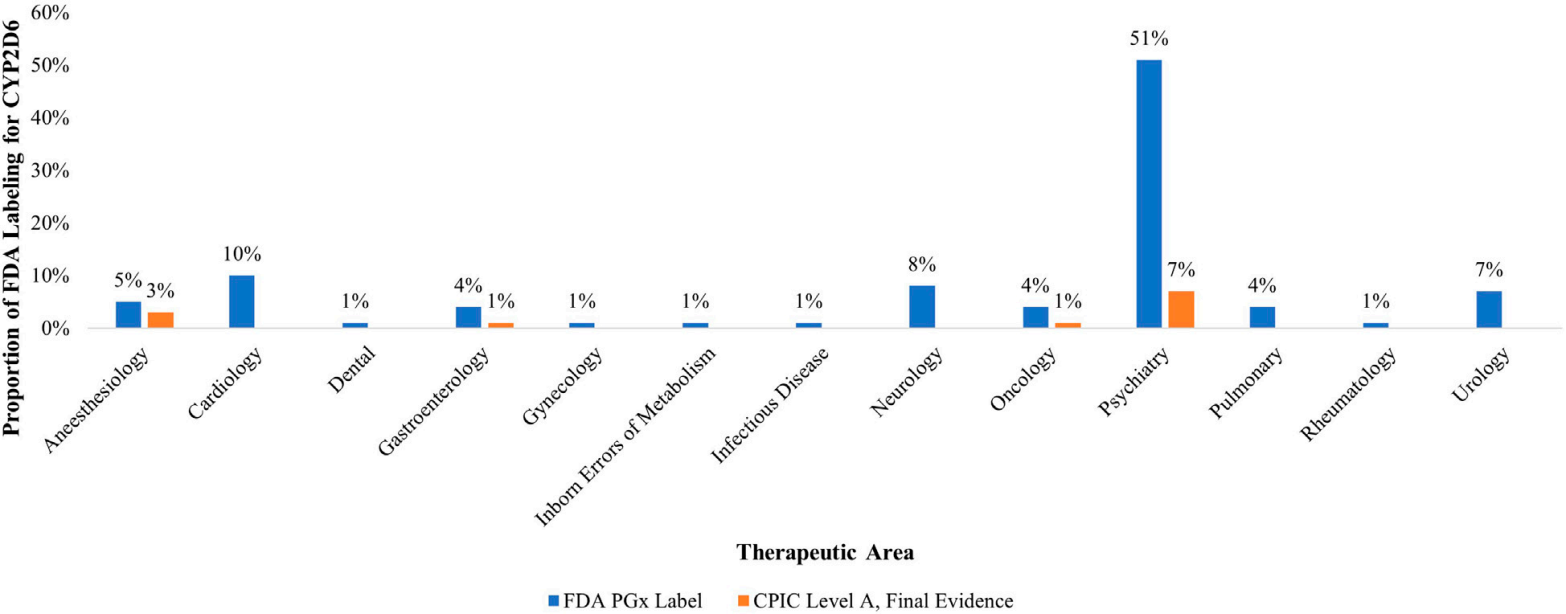
Proportion of therapeutic areas with US FDA labels for pharmacogenomic biomarker CYP2D6 and CPIC Level A, Final Evidence drugs (n = 73). Legend: This figure demonstrates both the regulatory and clinical significance of PGx biomarker *CYP2D6. CYP2D6* accounts for the highest proportion of all FDA PGx labeling sections (∼13%) and is disproportionately demonstrated in psychiatry (51%), followed by cardiology, neurology, and anesthesiology respectively. Of the thirteen therapeutic areas which have FDA PGx labeling sections, only four of these therapeutic areas also have CPIC Level A final evidence, illustrating misalignment between clinical guidance and regulatory acknowledgement.

Similarly, the U.S. Food and Drug Administration (FDA) Table of Pharmacogenomic Biomarkers in Drug Labeling associates *CYP2D6* with 73 different medications [6]. Of all biomarkers within the Table, and thus of regulatory concern, *CYP2D6* holds the highest number of labeling sections (∼13% of 570 total) [6]. As seen in Figure 1, *CYP2D6* holds special pharmacological potential and significance in psychiatry, a therapeutic area holding 50% of all its labeling sections, followed by cardiology (10%), neurology (8%), and urology (7%). Among the ten *CYP2D6*-drug pairs in CPIC, nine have FDA PGx drug labeling sections (see Figure 1).

Evidence is often generated through randomized, controlled clinical trials. However, such trials face structural and systemic challenges that interfere with accurate, generalizable, and efficient investigation of critical research questions spanning various therapeutic areas. Some of these challenges are limited trial availability, access barriers, and eligibility issues for prospective trial participants, especially racial/ethnic minorities and populations with comorbidities, contributing to systemic barriers that impede participation in research and institutional mistrust. In fact, a recent National Academies report showed that despite making up 39% of the U.S. population, historically underrepresent racial and ethnic groups represent between 2% and 16% of trial participants [7]. Underrepresentation becomes even more prevalent in cases where participant characteristics become even more diversified beyond race/ethnicity (i.e., sex/gender, pregnancy status, comorbidity status, and age).

To address these challenges, real-world evidence (RWE) on the clinical utility of *CYP2D6* biomarker testing across each of these therapeutic areas can be generated through the analysis of real-world data (RWD) deriving from a diverse array of data sources [8, 9]. This includes, but is not limited to, electronic health record (EHR) data and claims data, to help better characterize, clarify, and/or establish RWE on the therapeutic (un)importance of *CYP2D6* biomarker testing. Doing so among and across demographically diverse (sub)populations would be vitally important to maximize potential therapeutic benefit and reducing the likelihood of potential serious adverse events associated with biological variation in *CYP2D6*-mediated drug metabolism among patients. Regulators, payers, and health technology assessment (HTA) bodies leveraging insights from EHR and claims data to evaluate new and/or existing drugs across several therapeutic areas with *CYP2D6* biomarker implications also stand to benefit from understanding the therapeutic applications of *CYP2D6* biomarker testing in real-world settings.

To date, no systematic review has been published exploring the utility and impact of *CYP2D6* testing to anticipate drug-gene interactions in real-world clinical settings and with an intentional focus on such insights derived from EHR and/or claims data. Here, we present our review of such evidence to help inform regulators, payers/HTA bodies, clinicians, and patients or patient advocates.

## Methods

### PICO assessment

Our PICO assessment was as follows:

Patient: Patients of any age with or without PGx testing to guide therapeutic options or decisions, discernable from EHR and/or insurance claims data.

Intervention: *CYP2D6* PGx biomarker testing implementation among and across various types of health care service providers.

Comparison: Patients and health care service providers not undergoing *CYP2D6* PGx biomarker testing) were compared to those who underwent testing.

Outcome: PGx clinical implementation strategies, patient-level outcomes (e.g., improved well-being, disease progression), patient health behavior (e.g., medication adherence), and health care service provider treatment decisions following *CYP2D6* PGx testing.

### Search strategy

Four authors (RH-S, NE, PR, and EK) conducted a literature search in June 2024 for original articles or research published at any time in the English language and indexed in Google Scholar, PubMed, Scopus, and Semantic Scholar. No specific filters or search limits were applied. The following search strategies were used:

• cyp2d6 AND drug interaction AND (“electronic health record” OR “electronic medical record”)
• cyp2d6 AND drug interaction AND (“electronic health record” OR “electronic medical record”) AND “drug-gene interaction”
• cyp2d6 AND drug interaction AND (“electronic health record” OR “electronic medical record”) OR (“claims data” OR “administrative data”) AND “drug-gene interaction”

### Inclusion/exclusion criteria

Papers were included in our analysis if they presented, measured, and/or described real-world clinical or patient and/or provider behavioral outcomes based on an assessment of EHR or insurance claims data following PGx testing for the *CYP2D6* biomarker, included PGx biomarker(s)-drug pairs with CPIC Level A and Final Evidence designations, and were published in the English language (inclusion criteria). Papers were excluded if they were not published in the English language, did not present real-world clinical or patient and/or provider outcomes based on an assessment of EHR or insurance claims data following PGx testing for the *CYP2D6* biomarker, did not include PGx biomarker(s)-drug pairs with CPIC Level A and Final Evidence designations, contained duplicates, or were located online at broken links.

### Article screening and selection

One senior author (RH-S.) initially and manually reviewed a subset of article titles and abstracts to facilitate further review among the co-authors. Subsequent manual reviews were conducted by three authors (NE, PR, and EK) for all remaining articles to identify articles for inclusion/exclusion. In cases of uncertainty among the three authors (NE, PR, and EK) concerning inclusion/exclusion following their reviews of titles, abstracts, and/or full papers, a fourth author (RH-S) was consulted to render a final decision to include/exclude any article.

### Quality assurance assessment

The PRISMA checklist and reporting guideline was used to support our analysis and guide our reporting of studies evaluating the effects of *CYP2D6* PGx testing as an intervention (see Supplementary Figure S1). Due to the differences in study objective, design, and reporting strategies, two distinct tools were applied to assess study quality and risk of bias, each suited to different study types. The Newcastle-Ottawa Scale (NOS) was used for non-randomized studies [10]. The A comprehenSive tool to Support rEporting and critical appraiSal of qualitative, quantitative, and mixed methods implementation reSearch outcomes (ASSESS) tool was also used for the remaining studies that did not fit the NOS criteria [11]. This dual approach ensured that each study was evaluated using the most appropriate criteria based on its methodological design. While individual studies’ sample size information was extracted as part of data analysis, sample sizes were not used to evaluate evidence-strength of studies nor did evidence-strength formally impact studies’ individual influence on final recommendations.

### Evaluation of selected studies

We assessed studies both quantitatively (descriptive analysis using Microsoft Excel) through weighted average comparisons of extracted data elements, described below, and qualitatively (single layer, bottom-up topic modeling) to identify categorical themes collectively across all studies. Weighted, as opposed to unweighted, averages were used to account for variance in sample size across studies. Two authors (PR and EK) conducted an initial assessment of descriptive statistical information presented in each study. Those two authors developed categorical themes for discussion first with a third author (NE) following final discussion with a fourth author (RH-S). Themes and subthemes were refined until >95% agreement was reached among all four authors.

### Data extraction

The following data, when present, were extracted from each study:

• Author/year;
• Aims and purpose of the study;
• Drug-biomarker pairs along with associated drug classes and therapeutic areas;
• Genes researched in conjunction with the biomarker of interest;
• Number of participants within each study, including those who received PGx testing;
• Participant/patient *CYP2D6* metabolizer phenotype;
• Demographic information: gender/sex, age, race, ethnicity, and genetic ancestry;
• Clinical outcomes following PGx testing implementation;
• Provider and/or patient behavioral outcomes following PGx testing implementations;
• PGx qualitative themes and subthemes.

Three authors (NE, PR, and EK) extracted data from all studies that met the inclusion criteria. Where possible, we calculated weighted averages across extracted data to account for variance in study sample sizes. The accuracy and clarity of qualitative and quantitative findings from extracted data were then confirmed by two senior authors (RH-S and CL).

## Results

### Study selection and broad characteristics

**FIGURE 2:**
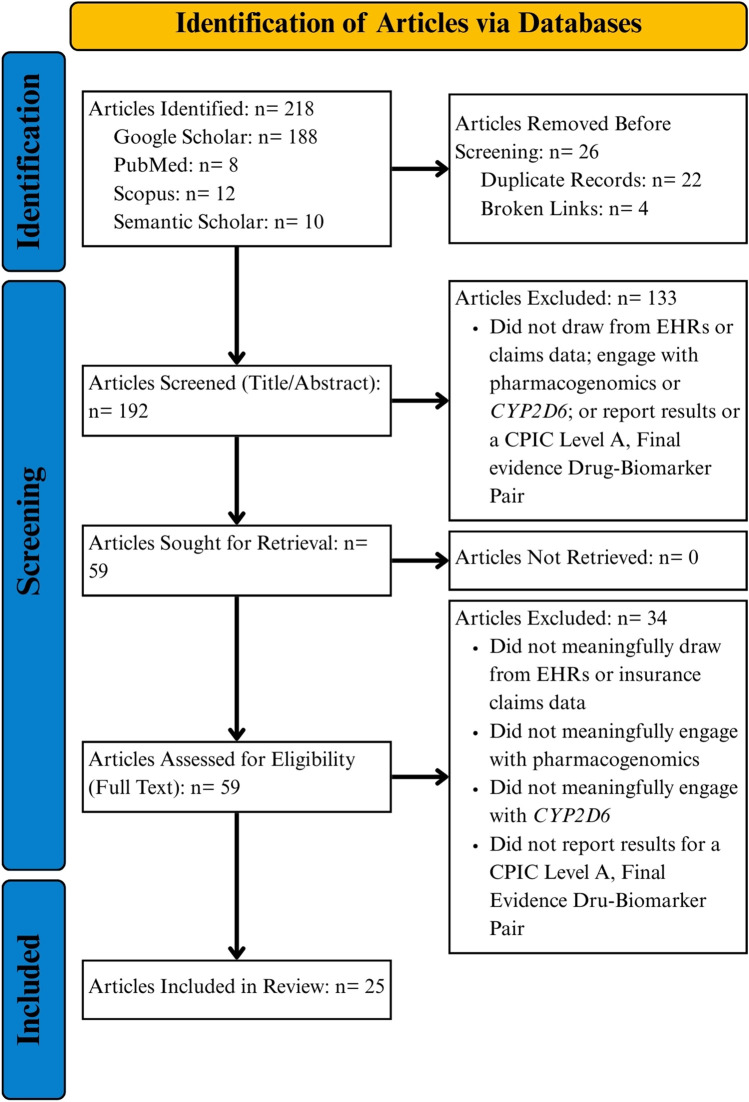
PRISMA flowchart.

We identified a total of 218 articles across Google Scholar (n = 188), PubMed (n = 8), Scopus (n = 12), and Semantic Scholar (n = 10; see PRIMSA flow diagram in Figure 2). Upon excluding duplicate articles and articles at broken links, 192 articles remained for initial title and abstract screening. A total of 133 articles were excluded after reviewing titles and abstracts for relevance to the study question, leaving 59 articles sought for retrieval and full text review. Following full text review, 33 articles were out of scope, leaving 25 for final analysis [12–36]. No study presented randomized controlled trial evidence. One study was open-label, non-randomized [23] and five were retrospective, observational in nature [22, 29, 31, 34, 35]. The remaining studies focused on lessons learned from implementing PGx into clinical systems or reported phenotype prevalence in different patient cohorts (e.g., managed care, advanced, cancer, etc.).

### Quality assessment of selected studies

NOS scores ranged from four to seven (maximum possible score of eight) with an average score of 6.4 across all selected studies. Two studies, Aka et al. and Bertholim-Nasciben et al., scored at 4 and were the only two studies scoring below 7 [12, 17]. This was due to point reductions on the Comparability (inadequate or lack of study controls) and Outcome (no long-term outcomes follow-up) metrics. ASSESS scores ranged from one to five (maximum possible score of five)—higher scores represent lower risk of bias—with an average score of 4.5. Individual study scores for NOS and ASSESS are available in the Supplemental (Supplementary Tables S1, S2).

### Overall quantitative and qualitative assessment

Across all selected studies quantitative data categories were metabolizer phenotypes (n = 10), gender/sex (n = 17), age (n = 14), race (n = 13), ethnicity (n = 4), genetic ancestry (n = 1), and PGx implementation outcomes (n = 7). Qualitative findings comprised of seven subthemes across two overarching themes. Theme 1 comprised of four (4) *CYP2D6-Specific* subthemes centered on genes researched alongside *CYP2D6* (n = 20), metabolizer phenotypes (n = 17), gene sequencing strategies (n = 9), and phenoconversion (n = 3). Theme 2 comprised of three *PGx and RWD Specific* subthemes centered on patient sub-groups in retrospective RWD analysis (n = 19), PGx implementation considerations (n = 8), and the range of health care provider decisions following PGx testing (n = 6). Ancillary quantitative and qualitative summaries for selected articles are provided the Supplementary Tables S3–S5.

### Identified drugs, therapeutic areas, and clinical outcomes

The foundational results from the analyzed literature are the drugs (and associated drug classes and therapeutic areas) implicated in *CYP2D6* PGx testing. Studies were summarized according to each drug, drug class, and therapeutic area associated with *CYP2D6* testing. Therapeutic areas were tabulated based on drugs and drug classes associated with *CYP2D6*, rather than all therapeutic areas mentioned within a given article.

*CYP2D6*-drug pairs with CPIC Level A, Final evidence were identified across studies selected for in our literature search across four therapeutic areas: analgesia (n = 21), psychiatry (n = 17), oncology (n = 7), and gastroenterology (n = 6). Nine (n = 9) drugs implicated in *CYP2D6* PGx testing were explored. Of these nine *CYP2D6*-drugs, eight had existing FDA PGx Biomarker labeling section. The two most researched drugs were codeine (n = 21) and tramadol (n = 18)—both opioids. Five drugs across three drug classes were within the therapeutic area of psychiatry: selective serotonin reuptake inhibitors (SSRIs, n = 11), tricyclic antidepressants (TCAs, n = 11), and selective norepinephrine reuptake inhibitor (SNRIs, n = 5). All other therapeutic areas had drugs belonging to one drug class (see Table 1). Atomoxetine, a psychiatric SNRI evaluated in five (5) studies, was the only drug without an FDA PGx Biomarker labeling section.

**TABLE 1:** *CYP2D6*-Impliacted Therapeutic Areas and Drug Classes Mentioned Across 25 studies published as of June 2024.

Therapeutic areas (n)	Drug classes (n)	Drugs (n)
Analgesia (21)	Opioid (21)	Codeine* (21)
		Tramadol* (18)
Psychiatry (17)	Selective Serotonin Reuptake Inhibitor (SSRI) (11)	Paroxetine* (11)
		Vortioxetine* (1)
	Tricyclic Antidepressant (TCA) (11)	Amitriptyline* (11)
		Nortriptyline* (8)
	Selective Norepinephrine Reuptake Inhibitor (SNRI) (5)	Atomoxetine (5)
Oncology (7)	Selective Estrogen Receptor Modulator (SERM) (7)	Tamoxifen* (7)
Gastroenterology (6)	5-HT3 Receptor Antagonist (6)	Ondansetron* (6)

Six (6) of 25 articles reported PGx clinical outcomes, considered to be a “measurable change in symptoms, overall health, ability to function, quality of life, or survival outcomes” in relation to PGx testing [37] Table 2). Of these six studies, three (3) focus on analgesia, with other investigated therapeutic areas being psychiatry and oncology. There was no consistent clinical outcome of interest investigated. All but one were observational, retrospective studies.

**TABLE 2:** Articles reporting PGx clinical outcomes.

Author	Study purpose	Outcome description	Study size	Study design	Therapeutic area
Chanfreau-Coffinier et al. [22]	Determining DGI risk among opioid users	Veterans with chronic opioid use were more likely to have higher care utilization. Veterans with chronic opioid use were more likely to receive CYP2D6-implicated medications	2,438,534	Observational, retrospective	Analgesia
Dorfman et al. [23]	Impact of PGx medication management	Following PGx testing, medications were changed and drug-therapy problems (DTPs) resolved	985	Open label, non-randomized	Psychiatry and Analgesia
Michaud et al. [29]	Assessment of PGx variants and medication risk scores impact on healthcare costs	High medication risk score with PGx variant increases risks of treatment failure, health care expenditure, and increased opioid doses	4,088	Observational, retrospective	Agnostic
Nichols et al. [31]	Calculates potentially avoidable adverse drug events with PGx	Several adverse events for ondansetron (nausea/vomiting), codeine (hypoanalgesia/sedation), and paroxetine (persistent depression)	193	Observational, retrospective	Oncology
St Sauver et al. [19]	Assessed *CYP2D6* phenotypes with adverse drug events	UM and PM patient were more likely to experience poor pain control and experience adverse symptoms, which disrupted treatment regimens and undercutting prescriptions	257	Observational, retrospective	Analgesia
Takahashi et al. [35]	Assessed hospitalization risk for UM *CYP2D6* phenotype	In a patient cohort, found 47% of UM were hospitalized compared to 30% NMs and 62% of UM visited emergency departments compared to 49%	929	Observational, retrospective	Agnostic

### Theme 1: variation in CYP2D6 biomarker testing and interpretation

Theme 1 comprises subthemes that are: 1.1, Single gene sequencing vs. gene panel sequencing for PGx analysis (n = 9); 1.2, Genes researched along with CYP2D6 (n = 20); 1.3, Metabolizer phenotypes and inconsistent phenotype categorization (n = 17); and 1.4, Drug-gene interactions (natural genotype) vs. drug-drug-gene interactions (phenoconversion) (n = 3).

#### Subtheme 1.1: single gene sequencing vs. gene panel sequencing for PGx analysis

Across nine (9) studies, investigators alternated between relying on single gene sequencing vs. gene panel sequencing for PGx analysis [13, 14, 16, 17, 22, 24–26, 33]. Seven studies relied solely on panel sequencing [14, 16, 17, 24–26, 33] and one only on single-gene sequencing [13]. The last studies reviewed a dataset with both kinds of tests, within which 48 of 90 tests were single-gene [22]. Some studies highlighted benefits and drawbacks of testing strategies. On one hand, single gene sequencing allowed for targeted assessment of individual genes, typically performed after adverse drug events had been observed. On the other hand, gene panels provided researchers with a broader understanding of participants’ overall PGx situation. Panels were most often performed as a part of pre-emptive—before adverse drug events occurred—PGx testing. When gene panels were used, other genes were researched along with *CYP2D6* because of PGx evidence within guidelines and/or literature, rather than because those genes happened to be on the same panel as *CYP2D6*.

#### Subtheme 1.2: biomarkers researched along with CYP2D6

Twenty (20) studies researched other biomarkers alongside *CYP2D6* [12–20, 22–26, 29–33, 36]. Across all studies, 33 distinct biomarkers were reported (see Table 3). Biomarker testing outcomes most frequently researched alongside *CYP2D6* were *CYP2C19* (n = 17), *CYP2C9* (n = 13), and *SLCO1B1* (n = 8). Across the 33 genes, Cytochrome P450 genes were researched more often (n = 20) than those outside the CYP family (n = 13).

**TABLE 3:** Genes Researched in Conjunction with *CYP2D6* across 25 studies published as of June 2024.

Other genes	*n*
*CYP2C19*	17
*CYP2C9*	13
*SLCO1B1*	8
*CYP3A5*	7
*CYP3A4*	6
*VKORC1*	6
*TPMT*	5
*HLA-B*	5
*CYP4F2*	3
*CYP1A2*	3
*DPYD*	3
*CYP2B6*	3
*NUDT15*	2
*CYP4F1*	2
Other biomarkers^a^	1

^a^ *ATM, F5, UGT1A1, CYP1A1, CYP2A13, CYP2A4, CYP2A6, CYP2C8, CYP2E1, CYP2J2, CYP2R1, CYP2S1, CYP2W1, CYP3A43, CYP3A7, HLA-A, SLC15A2, SLC22A2, SLCO2B1*.

#### Subtheme 1.3: metabolizer phenotypes and inconsistent phenotype categorization

Of the 25 studies, 17 [15, 17–19, 21–24, 26–28, 30, 31, 33–36] qualitatively discussed *CYP2D6* phenotypes and 10 [14, 17, 22, 23, 26–28, 33, 35, 36] provided quantitative data. There were four primary phenotype categories. Importantly, these categories represent general metabolizer phenotypes rather than specific ‘at-risk’ phenotypes for specific biomarker-drug pairs. Individuals with abnormally low drug metabolism levels were Poor Metabolizers (PM). Intermediate Metabolizers (IM) had higher drug metabolism than PMs but did not metabolize at normal levels. Those who did metabolize normally were Normal or Extensive Metabolizers (NM or EM)—this review uses the more common classification of NM. The last major phenotype category was Ultrarapid Metabolizer (UM), when individuals metabolized drugs at a much higher rate than normal.

Outside the common categorizations, two studies included three intermediary categories: poor-to-intermediate, intermediate-to-extensive, and extensive-to-ultrarapid [26, 35]. Other studies outlined a Rapid Metabolizer (RM) category but only one reported numbers and percentages for that category—3 of 91 participants (4.4%) [14]. Studies that recognized the RM phenotype, but did not present corresponding data, experienced technical limitations which prevented the analysis of copy number variants (CVNs) and could not identify or differentiate between UMs and RMs. Because RM data was only reported once within the literature, and other intermediary categories only twice, they are considered outlier categories.

Excluding outliers, we calculated weighted averages for PM, IM, NM, and UM groups among study groups (see Table 4). Using weighted averages accounted for the wide range of study sizes and mitigated outsized impacts from any one study. Approximately 92% of participants had normal or intermediate metabolism for *CYP2D6*-implicated drugs and 12% were either poor or ultrarapid metabolizers. Importantly, due to inconsistent reporting of metabolizer phenotype across studies, the phenotype percentages do not add up to 100%.

**TABLE 4:** Weighted average percentage of patients with each CYP2D6 phenotype across all drugs.

Phenotype	Percentage without Verma et al. [36]	Percentage with Verma et al. [36]	Impact of including Verma et al. [36]Δ
PM	4.9%	3.6%	−1.3%
IM	35.5%	24.9%	−10.6%
NM	56.8%	37.9%	−18.9%
UM	7.1%	Not applicable	Not applicable

Changes represented with a delta symbol.

Despite weighting, the total results were evaluated with and without findings from Verma et al., because 66.3% (n = 28,778) of participants in Verma et al. had incomplete phenotype data [36]. This percentage was calculated by combining three of the study’s *CYP2D6* result categories: 2,701 (6.2%) ambiguous results, 12,541 (28.9%) indeterminate results, and 13,536 (31.2%) participants with no results. When included, the missing data dramatically decreased the weighted average calculations for each metabolizer category (Table 4). The study did not report UM rates or any outlier categories.

#### Subtheme 1.4: drug-gene interactions (natural genotype) vs. drug-drug-gene interactions (phenoconversion)

DGIs are when a specific drug interacts with a gene by either inhibiting or inducing it [15, 28]. In this case, implicated drugs taken by individuals with a *CYP2D6* variant resulted in abnormal or adverse drug responses due to altered drug metabolism by the CYP2D6 protein. Alternatively, DDGIs occur when one drug inhibits or activates a given gene, altering the efficacy or safety profile of a second drug [22]. Importantly, an individual may not necessarily have a variant form of *CYP2D6* but, because of a DDGI, may phenotypically experience variant-like drug metabolism. This is also known as phenoconversion. We identified DGIs and DDGIs as important, but distinct, areas of PGx research and treatment. Because phenoconversion leads to genotypically wild-type patients expressing variant phenotypes, PGx testing was considered useful for clearly distinguishing between these two sub-groups. One study determined phenoconversion is responsible for a majority of PM phenotypes, reporting an increase by “at least 2-fold” in PMs “after considering phenoconversion” [14].

### Theme 2: PGx test implementation and data considerations

Studies along Theme 2 (n = 22) comprised of three (3) subthemes [12–20, 22, 25–36]. Theme 2.1 describes the range of healthcare provider decisions following PGx testing. These include accepting PGx-based recommendations generally, specific medication modifications, and no changes in prescribing behavior. Theme 2.2 addresses patient sub-groups in retrospective RWD analysis, identifying trends regarding three sub-group identifiers: gender and sex (2.2.1); race, ethnicity, and genetic ancestry (2.2.2); and age (2.2.3). Theme 2.3 focuses on results about PGx implementation into clinical care, decision-making, and EHRs. From this theme, three common barriers to PGx implementation were identified: data inoperability (2.3.1), cost and reimbursement (2.3.2), and limited PGx knowledge (2.3.3). Additionally, Subtheme 2.3.4 outlines different implementation strategies between pharmacists and physicians.

#### Subtheme 2.1: range of healthcare provider decisions following PGx testing

Six studies under Subtheme 2.1 described provider responses to PGx-based recommendations [13, 14, 16, 22, 24, 30]. All studies assessed at least one other gene in addition to *CYP2D6* and do not differentiate between them when discussing recommendations. Within the literature, “recommendations” were suggested clinical actions based on a patient’s PGx profile, their prescribed medications, and established PGx medical research and guidelines. If studies specified what kind of recommendation was given, it was for a medication change. In some studies, recommendations were pharmacist-provided, whereas other studies involved computer-assisted or -prompted recommendations based on pre-defined rules (e.g., CPIC guidelines for specific medications) [16]. A provider’s “acceptance” of a recommendation referred to whether or not they followed the clinical suggestion. The study involved an assessment of EHR and claims data before and after a given recommendation to determine if a physician accepted or did not accept a PGx recommendation. One study, which relied solely on insurance claims, noted this methodology limited their ability to definitively confirm “whether the result of the…test guided the treatment choice” [13].

Three studies provide quantitative data on general acceptance of PGx-based recommendations [14, 16, 30]. In total, 668 PGx-based recommendations were made and 595 (89.1%) were accepted (see Table 5). Two studies (n = 2) researched how PGx testing impacted medication change orders specifically [13, 22]. One reviewed claims data and compared when PGx tests were ordered, results received, and when patients filled their prescriptions [13]. Of the 1,059 patients, 540 (51%) filled their prescriptions before test results were delivered. The study reported that the clinicians did not rely on PGx results for the first round of prescriptions and used testing to inform future prescribing decisions. Another study investigated medication changes following PGx testing using EHR data [22]. Of the 90 *CYP2D6* tests identified, 34 (38%) involved provider-facilitated medication changes.

**TABLE 5:** Articles reporting proportion of acceptance of pharmacogenomic test-based recommendations.

Article title	Prescribing recommendations	Accepted (%)
Arwood et al. [14]	62	54 (87%)
Bain et al. [16]	436	388 (89%)
Mills & Massmann [30]	170	153 (90%)
Total:	668	595 (89.1)

Finally, one study associated the type of healthcare provider with the rate of PGx recommendation acceptance [30]. The investigators assessed pharmacists, physicians, and advanced practice providers and found 100% (23/23) of pharmacists, 91% (88/97) of physicians, and 84% (41/49) of advanced practitioners followed PGx recommendations.

#### Subtheme 2.2: patient sub-groups in retrospective RWD analysis

Patient demographic information varied widely, with many studies missing key demographic details. Of the 25 articles/studies included in our full-text analysis, 19 [13–18, 20, 22, 23, 25–29, 31, 33–36] contained at least some demographic information, which our team considered to include total study size, gender/sex, age, race and ethnicity, and genetic ancestry data. The most consistently reported demographic information across studies were total study size (n = 19), gender/sex (n = 17), and average participant age (n = 14). Study sizes ranged widely, from 30 participants to 2,436,654 [22, 27, 28]. The median participant group size was 985 (IQR = 20,157.5) with study-level sample sizes reported in Supplementary Table S5.

##### Subtheme 2.2.1

A lack of differentiation for gender and sex (sub-)categories existed within the literature (n = 17) [13–18, 20, 22, 25–29, 31, 33, 35, 36]. Most studies reported gender and sex-based information as a single category, though studies reported sex and gender interchangeably. Studies in general did not discuss possible differences between self-reported gender in EHRs and genetically determined sex. One study discarded biobank samples in the event “genetically defined sex and EHR reported sex” showed “discordance” [36].

Reviewing the gender/sex data, females (58%) were represented more than males (42%) within the literature. The greatest difference between females and males within a study, drawn from a sub-group of 90 *CYP2D6* analyzed participants, was 19 females (18%) and 74 males (82%) [22]. The smallest ratio disparity was 31,781 females (55.7%) and 25,277 males (44.3%) [33].

##### Subtheme 2.2.2

The second subtheme identifies a lack of racial, ethnic, and genetic ancestry data within RWD sources. The literature categorized racial demographics into the following groups: White or Caucasian, Black or African American, Asian or Asian American, Native Hawaiian/Pacific Islander, Biracial, or Unknown. Ethnic categories, when considered separately from race, were Hispanic/Latinx and Ashkenazi Jews. Thirteen (13) studies reported some form of self-reported racial/ethnic information, both qualitatively and quantitatively [14–18, 22, 25–28, 31, 33, 36]. The categories Native Hawaiian or Pacific Islander, Biracial, and Ashkenazi were only reported once; weighted averages for all other categories are reported below (Table 6). Like metabolizer phenotype calculations, racial data were reported inconsistently across studies and therefore do not equal 100% when added together.

**TABLE 6:** Weighted averages per racial and ethnic categories.

Racial or ethnic category	Weighted average
White	74.5%
Black or African American	10.2%
Asian or Asian American	1.8%
Native Hawaiian/Pacific Islander	0.1%
Unknown	13%
Hispanic	4.3%
Non-Hispanic	95.7%

Study participants were most often majority White or Caucasian with the exception of one study [15]. Six studies comprised of 85% or more data from White participants. An average of 13% participants, across 11 studies, had Unknown racial data. Black or African American was the next most represented racial group, although making up an average of only 10.2% of the study population. Notably, this calculation does not include demographic data from one study due to the fact that the study combined, without specific rationale, “Black or African American” with “Biracial” (i.e., only two categories, “White” and ‘Black or Biracial’, were reported [27]. The last reported racial categories were Asian or Asian American, 1.8% (n = 4) and Native Hawaiian or Pacific Islander, 0.1% (n = 1).

Four studies reported ethnicity data, which largely distinguished between Hispanic/Latinx and non-Hispanic/Latinx [14–16, 36]. An average of 4.3% of participants belonged to the category. One article reported Ashkenazi Jewish ethnic data—0.4% (146/36,511) of participants within the study made up this sub-group [15].

Genetic ancestry data was reported in one study [36]. Of the study’s 1,896,012 participants, 69.2% (n = 1,314,800) had European ancestry, 25.7% (n = 488,300) were African, 1.6% (n = 30,400) were East Asian, 1.3% (n = 24,700) were South Asian, 1.3% (n = 24,700) were Mixed American, and 0.9% (n = 17,100) of participants had Unknown genetic ancestry.

##### Subtheme 2.2.3

The third subtheme describes the relatively greater proportion of participants aged 65 or older and underrepresentation of participants under 18-years-old. While average age was consistently reported within the literature (n = 14), individual studies differed in reporting ‘average age’ as a mean or median calculation [13, 14, 16–18, 20, 22, 23, 25–27, 29, 31, 35]. The weighted mean age calculated by our team across studies was 49.4; the median was 53.6. Three studies had average ages of 65 or older, and five studies had averages below 50.

Three studies assessed pediatric patient populations in some capacity [12, 24, 32]. One research team expressed being “challenged by the lack of drug/gene evaluations in pediatric patients” and the need to draw conclusions from research based primarily on adults as the motivation for their investigation into pediatric populations [32]. Studies assessing pediatric populations called for addressing “an urgent unmet need in pediatrics to refine and develop precision actionable PGx guidance” [24]. However, because *CYP2D6*’s expression reaches stable “levels equivalent to adults during infancy,” one study emphasized focusing on possible impacts of *CYP2D6*-implicated drugs “used in children for different indications than in adults” [12].

#### Subtheme 2.3: PGx implementation efforts for clinical care, decision-making, and EHRs

Following findings on participant demographic information, our team explored PGx implementation efforts for clinical care, decision-making, and EHRs (Subtheme 2.3). Of the four subthemes, three identify different technological (2.3.1), financial (2.3.2), and knowledge (2.3.3) implementation barriers. Subtheme 2.3.4 outlines diverging strategies for which kind of provider—physicians or pharmacists—led a health system’s implementation effort.

##### Subtheme 2.3.1

When attempting to integrate PGx test results into patients’ medical records, data inoperability issues between PGx test results and EHRs commonly occurred. Five studies qualitatively addressed this subtheme [16, 20, 24, 25, 32]. The study discussed that PGx test results are most often returned as unstructured data, like PDFs. EHRs, on the other hand, operate using structured data. Due to mismatched data types, PGx test results often could not be directly nor automatically uploaded to EHR systems. This hurdle, studies reported, prevented providers from easily integrating test results into their clinical decision workflow. One health system contracted with an external software company to develop a program that transformed unstructured PGx data into a structured format to address this issue [24]. The study team developed a structured number system matched to specific metabolism levels and generated pre-alerts based on the severity of possible DGIs.

##### Subtheme 2.3.2

The second observed implementation challenge were high costs of PGx testing and testing reimbursement barriers. Four studies—all published during or after 2020—discussed this difficulty [14, 24, 25, 32]. These studies found PGx tests, particularly when those tests were pre-emptive, had limited insurance coverage. One study reported reimbursement issues as a barrier to PGx test utilization, but did not “have sufficient data to understand reimbursement based upon patient insurance” differences [24].

Two studies provided quantitative data on how insurance coverage affected patients’ willingness to receive PGx testing. One utilized an internal medicine clinic to study PGx clinical adoption [14]. The clinic was grant funded for the first 2 years of the clinic’s operation. During this time, 91 patients were seen, 84 of which were recommended for PGx testing. Of these, 77 followed through with testing and seven (8.5%) refused due to costs. In its third year of operation, the clinic began billing insurance and saw 36 patients. For the 33 patients who were recommended testing, eight (23%) declined any kind of testing and two (6%) participants sought external PGx testing. The second study followed 69 patients who were recommended PGx testing [25]. Here, 68 (98.6%) of patients received testing and only one (1.4%) refused. However, that one patient “refused testing due to costs”—the out-of-pocket expenses were generally $300 or less [25].

##### Subtheme 2.3.3

A general lack of PGx knowledge in clinicians and patients was the final identified implementation barrier, present in eight studies [16, 18, 20, 22–24, 26, 32]. Within healthcare provider study groups, clinicians often felt as if they did not have a deep or satisfactory understanding of pharmacogenomics. Limited provider knowledge resulted in diminished “engagement” with PGx testing from clinicians [26]. Regarding clinicians’ responsiveness to clinical decision support tools, providing “education on the scientific basis of pharmacogenetics” was “essential to improve the prescribing physicians’ understanding of the clinical significance of PGx alerts” [23]. When that education was integrated into clinical care at the point-of-care, those modules were “well received by clinicians and pharmacists” [21]. Only one study reported on patient understandings of PGx information. In this study, 33% of patients either understood their PGx results a little or not at all [19].

##### Subtheme 2.3.4

The last subtheme did not focus on challenges with implementing PGx testing into clinical care, but rather general implementation strategies. Out of nine studies, two kinds of methods emerged: centralized, pharmacist-led clinics and decentralized, physician-led prescription decision-making [14, 16, 19, 20, 23–25, 30, 32]. The first method involved establishing a clinic run by a pharmacist with a specialty in pharmacogenomics (n = 4) [14, 16, 25, 32]. Physicians could refer patients who they thought might benefit from PGx testing to the clinic. While individual study sites differed, clinics followed the same general protocol. First, patients would meet with the pharmacist for an initial assessment, where the pharmacist would review their medical history (from both the EHR and patient) to decide whether or not to recommend PGx testing. If recommended, patients would receive testing and meet with the pharmacists when the results were available. Often, both the patient and their original provider would attend this second meeting. The pharmacist would review the test results, discuss its implications on whatever medication(s) a patient was taking or may take in the future, and provide a recommendation. The patient’s physician would then decide whether or not to accept the recommendation. Pharmacists effectively provided clinician and patient education about “the applications of PGx in clinical practice” [16].

Despite overarching similarities, individual PGx clinics were structured and operated differently. Some had only one pharmacist on staff, while others had several. Others met with patients only, physicians only, or had both present. The last major difference in clinic structure was who was able to submit clinic referrals. Only one study noted that 46% (32 of 69) of patients referred themselves [25].

Five studies reported a decentralized PGx implementation approach, with physicians individually ordering PGx tests, interpreting the results themselves, and subsequently making treatment decisions [18, 20, 23, 24, 30]. Often, physicians were supported by PGx-based clinical decision-making software, which provided recommendations based on (1) guidance from established clinical frameworks or (2) patients’ individual test results.

## Discussion

Our study holds several key findings that will be useful and informative for clinicians, epidemiologists, health researchers and economists, and regulators in the field of evaluating RWE to contextualize the value of *CYP2D6* PGx testing. First, of the ten *CYP2D6*-drug pairs with strong and final (CPIC Level A, Final) evidence, nine (90%) were identified in our review; tropisetron, an oncology drug that helps with nausea and vomiting, was not identified [38]. Additionally, only one drug among those nine, atomoxetine (a psychiatric drug), did not have a corresponding FDA PGx Drug label. This demonstrates a strong emphasis on investigating drug-gene pairs with known PGx risks within RWE literature. And, as seen in the range of healthcare provider decisions following PGx testing theme, when presented with PGx recommendations for strong evidence pairs, providers most often incorporate those recommendations into their decision-making. Combined with the six studies that reported clinical outcomes, only nine of 25 studies had findings relevant to clinical settings [14, 16, 22, 23, 29–31, 34, 35]. The remaining studies largely focused on either lessons learned from implementing PGx into clinical systems or reported on phenotype prevalence in different patient cohorts (e.g., managed care, advanced cancer, etc.) [12, 13, 15, 17–21, 24–28, 32, 33, 36].

Second, most studies (21 of 25) focused on two drugs used in pain management, likely reflecting a heightened scrutiny due to the ongoing opioid crisis [12, 14–24, 26–29, 31, 33–36]. Pain management often involves complex medication regimens and PGx testing can identify genetic variations that affect drug metabolism, efficacy, and safety. Additionally, 17 studies explored five different drugs relevant to psychiatry, demonstrating a considerable interest in how PGx testing might enhance treatment strategies in this field [12–19, 21, 22, 24, 25, 31–33, 35, 36]. Given most drugs identified are within the therapeutic scope of psychiatry, our findings are of particular importance for this field as it struggles with issues like workforce shortages that impact ability to diagnose and treat psychiatric patients in need [39, 40]. Patients in psychiatry often endure extensive trial-and-error phases when prescribed medications, characterized by poorly managed symptoms and adverse drug reactions, until suitable medications and dosages are identified [41]. Hence, there is a demand for strategies that anticipate or mitigate these adverse reactions and streamline treatment processes. This knowledge holds crucial importance for populations currently underrepresented in clinical trials and/or RWE studies with either an ability or potential to guide treatment protocols for managing the mental health of diverse patient populations, including but not limited to early mental illness intervention.

Third, RWD/E can be an important tool to enable inclusion of subgroups traditionally underrepresented in research. US Census data reports 75.3% of the population is White, 13.7% is Black, 1.3% are American Indian and Alaska Native, 6.4% are Asian, and 0.3% are Native Hawaiian and Other Pacific Islander [42]. Compared to demographic data from our review, White participants were researched comparably to the broader population at 74.5%. At 10.2% of research participants, Black patients were researched at a somewhat lower level. Asian patients were researched roughly 3.5 times less than their broader population make up. Native Hawaiian or Pacific Islander patients’ research was similarly disproportionate. Native Americans and Alaskan Natives, who compose 1.3% of the population, were not researched in any identified study. Given White and Black patients close to their proportion in the broader U.S., evidence for these *CYP2D6-*drug pairs is likely generalizable. But, for other patient subgroups with limited (or no) representation within this review, evidence may not be generalizable. This can reinforce treatment inequities, perpetuate data bias resulting from overestimations, and further exclude subgroups from benefits of the knowledge and innovation derived from research. However, as mentioned above, key gender/sex and racial/ethnic data were often unavailable in datasets. Furthermore, we found that pediatric populations are underrepresented in PGx research; only 3 of 25 studies reported on this patient population. Effectively utilizing RWD/E in PGx requires mitigating this tension to develop more inclusive/generalizable findings. Further, research should be more inclusive of individuals with varied sex chromosomes regardless of gender. Additionally, genetic ancestry was only investigated once, despite large impacts on population pharmacogenomics findings [43]. Other research programs, like the U.S. All of Us Research Program, have revealed health disparities driven by genetic ancestry [44].

Fourth, researchers largely emphasized implementation outcomes from the clinician’s perspective—both in terms of PGx knowledge and response to PGx-based recommendations. This clinician perspective preference may blur the distinction between changes in prescriber behavior and real-world patient outcomes. While it is critical to understand how clinicians adopt and apply PGx test results, it is equally important to understand how patient outcomes may or may not improve. PGx guidelines need to be informed by evidence, and researchers must take those guidelines and see how they measure in real-world based on both clinician and patient experiences with PGx testing. Another way to bridge this gap is for research to focus on patient-centered real-world outcomes, such as changes in pain levels, quality of life, or incidence of adverse drug reactions following the implementation of new practices.

To move the field forward, research subsidies can be helpful to address patient expenses associated with PGx testing. As seen in our review, despite increased coverage of PGx testing, research participants vary in responses to PGx testing costs before and after grant funding ends with rates of commercial and no testing increasing after grant funding runs out [45]. Identified studies were carried out in clinical settings, which already holds cost barriers, rather than strictly research settings. By providing financial support for research into PGx testing, subsidies can help cover these costs, in turn making the testing more affordable for patients. This financial assistance can enable broader implementation of PGx testing in clinical settings, ensuring that more patients benefit from personalized medicine approaches. It also encourages further research and development in the field, potentially leading to innovations that could reduce the cost of testing over time.

Despite the recognized benefits of improving sub-group representation in RWE research, these findings highlight persistent gaps in engaging traditionally underrepresented communities. Future RWE studies on *CYP2D6* and other pharmacogenomic biomarkers should be done in partnership with communities that are engaged in work focused on 1) improving the translation of study results across patient communities, 2) exploring the effect of *CYP2D6* biomarker testing to help address priority health concerns across one or more therapeutic areas described herein, and/or 3) building community-based participatory precision medicine research training or capacity. In fact, meaningful, trustworthy, and sustained community engagement across the RWE study development and implementation process will be critical to help characterize previously unknown *CYP2D6* variants of uncertain or undefined significance, such as those identified within the All of Us Research Program [46].

There are limitations to our study and findings. Given the *CYP2D6* phenotype is reliant on copy-number and structural variation and there are certain CYP2D6 star-alleles that do not define or include structural variation or a gene deletion, our findings do not provide such insight across the therapeutic areas noted herein [36]. Future work should continue to address this limitation by considering published work on this topic [47–49]. The Pharmacogene Variation Consortium, or PharmVar, for example, offers usable nomenclature and summaries describing structural variations of human *CYP2D6*, as well as recommended terms and definitions for clinical and research reporting, which can be useful to support ongoing work [50]. Additionally, key demographic data were reported inconsistently within the literature, leading to cumulative averages for racial and metabolizer phenotype data not equaling 100%. This represents a broader need for consistency in demographic reporting. A third limitation is that, upon inclusion in the review, the strength of evidence presented in each study (e.g., randomized control trial vs. observational, or small vs. large sample size) was not systematically differentiated or measured. Future studies should explicitly categorize studies based on evidence strength and assess how evidence strength influences author-proposed recommendations. Lastly, future research should employ more comprehensive keyword strategies (e.g., including variations such as “claims database” and “claims dataset”) to ensure broader capture of relevant literature.

Our findings can be used to substantiate the need for ongoing policy efforts intended to advance pharmacogenomic research and testing in real-world settings, like the most recent bipartisan legislation in the US called the Right Drug Dose Now Act [51]. The Act intends to accomplish the following key goals: 1) “update the National Action Plan for Adverse Drug Event Prevention by integrating advancements in pharmacogenomic research and testing,” and 2) enhance EHRs “with pharmacogenomic information to improve patient care and reduce adverse drug events” [51]. Therefore, such policy efforts hold significant value and serve as critical steps to help drive RWE research focused on *CYP2D6* and other pharmacogenomic biomarkers implicated across a range of therapeutic areas.

### Conclusion

Pharmacogenomic testing and research represents a beneficial way to advance precision medicine initiatives and improve patient care. Our work demonstrates that despite challenges with and varied strategies for implementing PGx into routine clinical care, when implemented, PGx testing for *CYP2D6*-drug pairs with high, established evidence can successfully support providers in their decision-making processes. Additionally, EHRs and insurance claims are useful data sources for establishing PGx testing effectiveness in real-world settings. Based on our findings, we recommend expanding patient sub-group analyses in future research to improve generalizability, exploring patient-centered real-world outcomes to better contextualize PGx-informed treatment, and investigating the effectiveness of PGx testing where evidence is limited or conflicting for *CYP2D6* and associated pharmaceuticals.

## Data Availability

The original contributions presented in the study are included in the article/Supplementary Material, further inquiries can be directed to the corresponding author.
