# Supplementary material for: A systematic review of real-world evidence on the clinical relevance, characterization, and utility of CYP2D6 biomarker testing
Source: J Pharm Pharm Sci. 2025 Aug 29;28:14708. doi: 10.3389/jpps.2025.14708 (PMC12425829; doi:10.3389/jpps.2025.14708)
Supplement: Supplementary file 1 [file Supplementaryfile1.docx]

Supplemental Materials for *A Systematic Review of Real-World Evidence on the Clinical Relevance, Characterization, and Utility of Pharmacogenomic CYP2D6 Biomarker Testing*

*Supplemental Figure 1.* PRISMA Checklist

| **Section and Topic** | **Item #** | **Checklist item** | **Location where item is reported** |
| --- | --- | --- | --- |
| **TITLE** | | |  |
| Title | 1 | Identify the report as a systematic review. | Title Page |
| **ABSTRACT** | | |  |
| Abstract | 2 | See the PRISMA 2020 for Abstracts checklist. | Abstract |
| **INTRODUCTION** | | |  |
| Rationale | 3 | Describe the rationale for the review in the context of existing knowledge. | Introduction, ¶4 |
| Objectives | 4 | Provide an explicit statement of the objective(s) or question(s) the review addresses. | Introduction, ¶4 |
| **METHODS** | | |  |
| Eligibility criteria | 5 | Specify the inclusion and exclusion criteria for the review and how studies were grouped for the syntheses. | Methods, ¶3 |
| Information sources | 6 | Specify all databases, registers, websites, organisations, reference lists and other sources searched or consulted to identify studies. Specify the date when each source was last searched or consulted. | Methods, ¶2 |
| Search strategy | 7 | Present the full search strategies for all databases, registers and websites, including any filters and limits used. | Methods, ¶2 |
| Selection process | 8 | Specify the methods used to decide whether a study met the inclusion criteria of the review, including how many reviewers screened each record and each report retrieved, whether they worked independently, and if applicable, details of automation tools used in the process. | Methods, ¶4 |
| Data collection process | 9 | Specify the methods used to collect data from reports, including how many reviewers collected data from each report, whether they worked independently, any processes for obtaining or confirming data from study investigators, and if applicable, details of automation tools used in the process. | Methods, ¶4 – 7 |
| Data items | 10a | List and define all outcomes for which data were sought. Specify whether all results that were compatible with each outcome domain in each study were sought (e.g. for all measures, time points, analyses), and if not, the methods used to decide which results to collect. | Methods, ¶4 – 7 |
|  | 10b | List and define all other variables for which data were sought (e.g. participant and intervention characteristics, funding sources). Describe any assumptions made about any missing or unclear information. | Methods, ¶4 – 7 |
| Study risk of bias assessment | 11 | Specify the methods used to assess risk of bias in the included studies, including details of the tool(s) used, how many reviewers assessed each study and whether they worked independently, and if applicable, details of automation tools used in the process. | Methods, ¶5 |
| Effect measures | 12 | Specify for each outcome the effect measure(s) (e.g. risk ratio, mean difference) used in the synthesis or presentation of results. | Methods, ¶5 |
| Synthesis methods | 13a | Describe the processes used to decide which studies were eligible for each synthesis (e.g. tabulating the study intervention characteristics and comparing against the planned groups for each synthesis (item #5)). | Methods, ¶4 – 7 |
|  | 13b | Describe any methods required to prepare the data for presentation or synthesis, such as handling of missing summary statistics, or data conversions. | Methods, ¶4 - 7 |
|  | 13c | Describe any methods used to tabulate or visually display results of individual studies and syntheses. | Methods, ¶4 – 7 |
|  | 13d | Describe any methods used to synthesize results and provide a rationale for the choice(s). If meta-analysis was performed, describe the model(s), method(s) to identify the presence and extent of statistical heterogeneity, and software package(s) used. | Methods, ¶4 – 7 |
|  | 13e | Describe any methods used to explore possible causes of heterogeneity among study results (e.g. subgroup analysis, meta-regression). | Methods, ¶4 – 7 |
|  | 13f | Describe any sensitivity analyses conducted to assess robustness of the synthesized results. | Not reported |
| Reporting bias assessment | 14 | Describe any methods used to assess risk of bias due to missing results in a synthesis (arising from reporting biases). | Methods, ¶5 |
| Certainty assessment | 15 | Describe any methods used to assess certainty (or confidence) in the body of evidence for an outcome. | Methods, ¶5 – 6 |
| **RESULTS** | | |  |
| Study selection | 16a | Describe the results of the search and selection process, from the number of records identified in the search to the number of studies included in the review, ideally using a flow diagram. | Results, ¶1 |
|  | 16b | Cite studies that might appear to meet the inclusion criteria, but which were excluded, and explain why they were excluded. | Not reported |
| Study characteristics | 17 | Cite each included study and present its characteristics. | Supplemental Tables 3 - 5 |
| Risk of bias in studies | 18 | Present assessments of risk of bias for each included study. | Results, ¶2; Supplemental Tables 1 - 2 |
| Results of individual studies | 19 | For all outcomes, present, for each study: (a) summary statistics for each group (where appropriate) and (b) an effect estimate and its precision (e.g. confidence/credible interval), ideally using structured tables or plots. | Not reported |
| Results of syntheses | 20a | For each synthesis, briefly summarise the characteristics and risk of bias among contributing studies. | Results, ¶1 – 3 |
|  | 20b | Present results of all statistical syntheses conducted. If meta-analysis was done, present for each the summary estimate and its precision (e.g. confidence/credible interval) and measures of statistical heterogeneity. If comparing groups, describe the direction of the effect. | Results, ¶4 – 35 |
|  | 20c | Present results of all investigations of possible causes of heterogeneity among study results. | Results, ¶4 – 35 |
|  | 20d | Present results of all sensitivity analyses conducted to assess the robustness of the synthesized results. | Not reported |
| Reporting biases | 21 | Present assessments of risk of bias due to missing results (arising from reporting biases) for each synthesis assessed. | Results, ¶4 – 35 |
| Certainty of evidence | 22 | Present assessments of certainty (or confidence) in the body of evidence for each outcome assessed. | Results, ¶4 – 35 |
| **DISCUSSION** | | |  |
| Discussion | 23a | Provide a general interpretation of the results in the context of other evidence. | Discussion, ¶1 – 6 |
|  | 23b | Discuss any limitations of the evidence included in the review. | Discussion, ¶7 |
|  | 23c | Discuss any limitations of the review processes used. | Discussion, ¶7 |
|  | 23d | Discuss implications of the results for practice, policy, and future research. | Discussion, ¶8; Conclusion, ¶1 |
| **OTHER INFORMATION** | | |  |
| Registration and protocol | 24a | Provide registration information for the review, including register name and registration number, or state that the review was not registered. | Not reported |
|  | 24b | Indicate where the review protocol can be accessed, or state that a protocol was not prepared. | Not prepared |
|  | 24c | Describe and explain any amendments to information provided at registration or in the protocol. | Not reported |
| Support | 25 | Describe sources of financial or non-financial support for the review, and the role of the funders or sponsors in the review. | Funding |
| Competing interests | 26 | Declare any competing interests of review authors. | Not reported |
| Availability of data, code and other materials | 27 | Report which of the following are publicly available and where they can be found: template data collection forms; data extracted from included studies; data used for all analyses; analytic code; any other materials used in the review. | Not reported |

*From:* Page MJ, McKenzie JE, Bossuyt PM, Boutron I, Hoffmann TC, Mulrow CD, et al. The PRISMA 2020 statement: an updated guideline for reporting systematic reviews. BMJ 2021;372:n71. doi: 10.1136/bmj.n71

| *Supplemental Table 1.* Newcastle Ottawa Scale - Study Quality Assessment | | | | |
| --- | --- | --- | --- | --- |
| Article Title (Year) | Selection | Comparability | Outcome | Total Score (mean = 6.4) |
| Aka et al. (2017) | *** |  | * | 4 |
| Anderson et al. (2022) | *** | * | *** | 7 |
| Ashcraft et al. (2022) | *** | * | ** | 6 |
| Bertholim-Nasciben et al. (2023) | *** |  | * | 4 |
| Bielinksi et al. (2014) | *** | * | *** | 7 |
| Bielinksi et al. (2020) | *** | * | *** | 7 |
| Chanfreau-Coffinier et al. (2022) | *** | * | *** | 7 |
| Dorfman et al. (2020) | *** | * | *** | 7 |
| Ji et al. (2016) | *** | * | *** | 7 |
| Knisely et al. (2017) | *** |  | *** | 6 |
| Michaud et al. (2021) | *** | * | *** | 7 |
| Nichols et al. (2019) | *** | * | *** | 7 |
| Pasternak et al. (2022) | *** |  | *** | 6 |
| St Sauver et al. (2017) | *** | * | *** | 7 |
| Takahashi et al. (2017) | *** | * | *** | 7 |
| Verma et al. (2022) | *** |  | *** | 6 |

| *Supplemental Table 2.* ASSESS Tool - Implementation Study Quality Assessment | | | | | | | |
| --- | --- | --- | --- | --- | --- | --- | --- |
| Qualitative Studies | | Criteria and Assessment | | | | | |
| Article Title (Year) | Meets general ASSESS expectations | Is the qualitative approach appropriate to answer the research question? | Are the qualitative data collection methods adequate to address the research question? | Are the findings adequately derived from the data? | Is the interpretation sufficiently substantiated by data? | Is there coherence between qualitative data sources, collection, analysis and interpretation? | Degree of Bias |
| Gill et al. (2021) | * | 1 | 1 | 1 | 1 | 1 | 5 |
| Paetznick et al. (2022) | * | 1 | 1 | 1 | 1 | 1 | 5 |
| Quantitative, Non-randomized Studies | | Criteria and Assessment | | | | | |
| Article Title (Year) | Meets general ASSESS expectations | Are the participants representative of the target population? | Are measurements appropriate regarding both the outcome and intervention (or exposure)? | Are there complete outcomes data? | Are the confounders accounted for in the design and analysis? | During the study period, is the intervention administered (or exposure occurred) as intended? | Degree of Bias |
| Arwood et al. (2020) | * | 1 | 1 | 1 | 0 | 1 | 4 |
| Caraballo et al. (2017) |  | 0 | 1 | 1 | 0 | 1 | 3 |
| Mills & Massmann (2023) | * | 1 | 1 | 1 | 0 | 1 | 4 |
| Mixed Methods Studies | | Criteria and Assessment | | | | | |
| Article Title (Year) | Meets general ASSESS expectations | Is there an adequate rationale for using a mixed methods design to address the research question? | Are the different components of the study effectively integrated to answer the research question? | Are the outputs of the integration of qualitative and quantitative components adequately interpreted? | Are divergences and inconsistencies between quantitative and qualitative results adequately addressed? | Do the different components of the study adhere to the quality criteria of each tradition of the methods involved? | Degree of Bias |
| Bain et al. (2018) | * | 1 | 1 | 1 | 1 | 1 | 5 |
| Caraballo et al. (2015) | * | 1 | 1 | 1 | 1 | 1 | 5 |
| Hoffecker et al. (2023) | * | 1 | 1 | 1 | 1 | 1 | 5 |
| An asterisk (*) indicates an article met the ASSESS tool's general expectations. For the 'Mixed Methods Criteria and Assessment' section, a "0" indicates a criterium was not met and a "1" means that criterium was met. Results are summed and presented as an article's degree of bias. Higher scores (3 - 5) are considered lower bias risk and lower scores (1 - 2) are considered higher bias risk. | | | | | | | |

| *Supplemental Table 3.* Theme 1, *CYP2D6* Biomarker specific, Articles by Theme | | | | |
| --- | --- | --- | --- | --- |
| Article Title (Year) | Subtheme 1: Single Gene Sequencing vs. Gene Panel Sequencing for PGx Analysis | Subtheme 2: Genes Researched Along with *CYP2D6* | Subtheme 3: Metabolizer Phenotypes and Inconsistent Phenotype Categorization | Subtheme 4: Natural Genotype (DGIs) vs Phenoconversion (DDGIs) |
| Aka et al. (2017) | NR | X | NR | NR |
| Anderson et al. (2022) | X | X | NR | NR |
| Arwood et al. (2020) | X | X | NR | NR |
| Ashcraft et al. (2022) | NR | X | X | X |
| Bain et al. (2018) | X | X | NR | NR |
| Bertholim-Nasciben et al. (2023) | X | X | X | NR |
| Bielinksi et al. (2014) | NR | X | X | NR |
| Bielinksi et al. (2020) | NR | X | X | NR |
| Caraballo et al. (2015) | NR | X | NR | NR |
| Caraballo et al. (2017) | NR | NR | X | NR |
| Chanfreau-Coffinier et al. (2022) | X | X | X | X |
| Dorfman et al. (2020) | NR | X | X | NR |
| Gill et al. (2021) | X | X | X | NR |
| Hoffecker et al. (2023) | X | X | NR | NR |
| Ji et al. (2016) | X | X | X | NR |
| Knisely et al. (2017) | NR | NR | X | X |
| Knisely et al. (2018) | NR | NR | X | NR |
| Michaud et al. (2021) | NR | X | NR | NR |
| Mills & Massmann (2023) | NR | X | X | NR |
| Nichols et al. (2019) | NR | X | X | NR |
| Paetznick et al. (2022) | NR | X | NR | NR |
| Pasternak et al. (2022) | X | X | X | NR |
| St Sauver et al. (2017) | NR | NR | X | NR |
| Takahashi et al. (2017) | NR | NR | X | NR |
| Verma et al. (2022) | NR | X | X | NR |
| Total Articles | 9 | 20 | 17 | 3 |
| X = an article possessed qualitative data (or data used for qualitative analysis) for a given category. NR = not reported, an article did not possess qualitative data (or data used for qualitative analysis) for a given category | | | | |

| *Supplemental Table 4.* Theme 2, PGx and RWD/E specific, Articles by Theme | | | | | | | | |  |  |
| --- | --- | --- | --- | --- | --- | --- | --- | --- | --- | --- |
| Article Title (Year) | Subtheme 2.1: Range of Healthcare Provider Decisions Following PGx Testing | Subtheme 2.2: Patient Sub-Groups in Retrospective RWD Analysis (n = 19) | | | Subtheme 2.3: PGx Implementation in Clinical Care, Decision-Making, and EHRs (n = 8) | | | |  |  |
|  |  | Subtheme 2.2.1: Lack of Differentiation for Gender and Sex (Sub-Categories) | Subtheme 2.2.2: Lack of Racial, Ethnic, and Genetic Ancestry Data | Subtheme 2.2.3: Relatively Greater Proportion of Participants Age 65 or Older | Subtheme 2.3.1: Data Inoperability Between PGx Results and EHRs | Subtheme 2.3.2: Cost and Reimbursement Barriers for PGx Tests | Subtheme 2.3.3: General Lack of PGx Knowledge in Clinician and Patient (Sub-)Groups | Subtheme 2.3.4: Centralized, Pharmacist-led Clinics and Decentralized, Physician-led Prescription Decision-Making |  |  |
| Aka et al. (2017) | NR | NR | NR | X | NR | NR | NR | NR |  |  |
| Anderson et al. (2022) | X | X | NR | X | NR | NR | NR | NR |  |  |
| Arwood et al. (2020) | X | X | X | X | NR | X | NR | X |  |  |
| Ashcraft et al. (2022) | NR | X | X | NR | NR | NR | NR | NR |  |  |
| Bain et al. (2018) | X | X | X | X | X | NR | X | X |  |  |
| Bertholim-Nasciben et al. (2023) | NR | X | X | X | NR | NR | NR | NR |  |  |
| Bielinksi et al. (2014) | NR | X | X | X | NR | NR | X | NR |  |  |
| Bielinksi et al. (2020) | NR | X | NR | NR | NR | NR | NR | X |  |  |
| Caraballo et al. (2015) | NR | X | NR | X | X | NR | X | X |  |  |
| Caraballo et al. (2017) | NR | NR | NR | NR | NR | NR | NR | NR |  |  |
| Chanfreau-Coffinier et al. (2022) | X | X | X | X | NR | NR | X | NR |  |  |
| Dorfman et al. (2020) | NR | NR | NR | X | NR | NR | X | X |  |  |
| Gill et al. (2021) | X | NR | NR | X | X | X | X | X |  |  |
| Hoffecker et al. (2023) | NR | X | X | X | X | X | NR | X |  |  |
| Ji et al. (2016) | NR | X | X | X | NR | NR | X | NR |  |  |
| Knisely et al. (2017) | NR | X | X | X | NR | NR | NR | NR |  |  |
| Knisely et al. (2018) | NR | X | X | NR | NR | NR | NR | NR |  |  |
| Michaud et al. (2021) | NR | X | NR | X | NR | NR | NR | NR |  |  |
| Mills & Massmann (2023) | X | NR | NR | NR | NR | NR | NR | X |  |  |
| Nichols et al. (2019) | NR | X | X | X | NR | NR | NR | NR |  |  |
| Paetznick et al. (2022) | NR | NR | NR | X | X | X | X | X |  |  |
| Pasternak et al. (2022) | NR | X | X | NR | NR | NR | NR | NR |  |  |
| St Sauver et al. (2017) | NR | X | NR | NR | NR | NR | NR | NR |  |  |
| Takahashi et al. (2017) | NR | X | NR | X | NR | NR | NR | NR |  |  |
| Verma et al. (2022) | NR | X | X | NR | NR | NR | NR | NR |  |  |
| Total Articles | 6 | 19 | 13 | 17 | 5 | 4 | 8 | 9 |  |  |
| X = an article possessed qualitative data (or data used for qualitative analysis) for a given category. NR = not reported, an article did not possess qualitative data (or data used for qualitative analysis) for a given category | | | | | | | | |  |  |

| *Supplemental Table 5*. Quantitative Data Analysis, Articles by Theme | | | | | | | | | | | |  |
| --- | --- | --- | --- | --- | --- | --- | --- | --- | --- | --- | --- | --- |
| Article Title (Year) | Therapeutics, Classes, Drugs | Other Genes Researched | Metabolizer Phenotype | Total Sample Size (n) | Gender/Sex | Age | Racial Data | Ethnic Data | Genetic Ancestry | PGx Implementation Outcomes |  | |
| **Aka** et al. (2017) | X | X | NR | NR | NR | NR | NR | NR | NR | NR |  | |
| Anderson et al. (2022) | X | X | NR | X (438,534) | X | X | NR | NR | NR | X |  | |
| **Arwood** et al. (2020) | X | X | X | X (91) | X | X | X | X | NR | X |  | |
| **Ashcraft** et al. (2022) | X | X | NR | X (36,511) | X | NR | X | X | NR | NR |  | |
| **Bain** et al. (2018) | X | X | NR | X (296) | X | X | X | X | NR | X |  | |
| **Bertholim**-Nasciben et al. (2023) | X | X | X | X (1,171) | X | X | X | NR | NR | NR |  | |
| **Bielinksi** et al. (2014) | X | X | NR | X (1013) | X | X | X | NR | NR | NR |  | |
| **Bielinksi** et al. (2020) | X | X | NR | NR | NR | NR | NR | NR | NR | NR |  | |
| **Caraballo** et al. (2015) | X | X | NR | X (67) | X | X | NR | NR | NR | NR |  | |
| **Caraballo** et al. (2017) | X | NR | NR | NR | NR | NR | NR | NR | NR | NR |  | |
| **Chanfreau**-Coffinier et al. (2022) | X | X | X | X (2,438,534) | X | X | X | NR | NR | X |  | |
| **Dorfman** et al. (2020) | X | X | X | X (985) | NR | X | NR | NR | NR | NR |  | |
| **Gill** et al. (2021) | X | X | NR | NR | NR | NR | NR | NR | NR | X |  | |
| Hoffecker et al. (2023) | X | X | NR | X (69) | X | X | X | NR | NR | X |  | |
| **Ji** et al. (2016) | X | X | X | X (1,013) | X | X | X | NR | NR | NR |  | |
| **Knisely** et al. (2017) | X | NR | X | X (30) | X | X | X | NR | NR | NR |  | |
| **Knisely** et al. (2018) | X | NR | X | X (30) | X | NR | X | NR | NR | NR |  | |
| **Michaud** et al. (2021) | X | X | NR | X (4,088) | X | X | NR | NR | NR | NR |  | |
| Mills & Massmann (2023) | X | X | NR | NR | NR | NR | NR | NR | NR | X |  | |
| **Nichols** et al. (2019) | X | X | NR | X (193) | X | X | X | NR | NR | NR |  | |
| Paetznick et al. (2022) | X | X | NR | NR | NR | NR | NR | NR | NR | NR |  | |
| **Pasternak** et al. (2022) | X | X | X | X (57,058) | X | NR | X | NR | NR | NR |  | |
| St **Sauver** et al. (2017) | X | NR | NR | X (257) | NR | NR | NR | NR | NR | NR |  | |
| **Takahashi** et al. (2017) | X | NR | X | X (929) | X | X | NR | NR | NR | NR |  | |
| **Verma** et al. (2022) | X | X | X | X (1,896,012) | X | NR | X | X | X | NR |  | |
| Total Articles | 25 | 20 | 10 | 19 | 17 | 14 | 13 | 4 | 1 | 7 |  | |
| X = an article possessed quantitative data (or data used for quantitative analysis) for a given category. NR = not reported, an article did not possess quantitative data (or data used for quantitative analysis) for a given category | | | | | | | | | | |  | |
